# Diagnostic and Therapeutic Approaches to Jaw Osteoradionecrosis

**DOI:** 10.3390/diagnostics14232676

**Published:** 2024-11-27

**Authors:** Yufan Wang, Heba Turkstani, Afrah Alfaifi, Sunday O. Akintoye

**Affiliations:** 1 Department of Oral and Maxillofacial Surgery, Peking University Shenzhen Hospital, Shenzhen 518036, China; wangyufan_1989@126.com; 2 Department of Oral Medicine, School of Dental Medicine, University of Pennsylvania, Philadelphia, PA 19104, USA; hebatur@upenn.edu (H.T.); alfaifia@upenn.edu (A.A.)

**Keywords:** osteoradionecrosis, diagnosis, therapy, stem cells, jaw, radiation

## Abstract

Jaw osteoradionecrosis (ORN) is a major complication of head and neck cancer radiotherapy. Treatment complications account for most of the poor outcomes for head and neck cancers and the associated racial health disparities in cancer survivorship. The global incidence of jaw ORN is improving due to pre-radiotherapy patient preparations and improved head and neck cancer radiotherapy protocols. The diagnosis and management of jaw ORN are based on the patient’s history and clinical presentation combined with radiological and histopathological tests. Evidence-based jaw ORN therapies focus on preventive, palliative, and surgical principles. However, new and innovative therapeutic approaches based on the cellular and molecular pathophysiological processes of jaw ORN and the jawbone’s susceptibility to radiation bone damage are limited. The rationale for this narrative review is to highlight the current diagnostic approaches to jaw ORN and the pathophysiological basis for new therapeutic options for ORN.

## 1. Introduction

**Figure 1:**
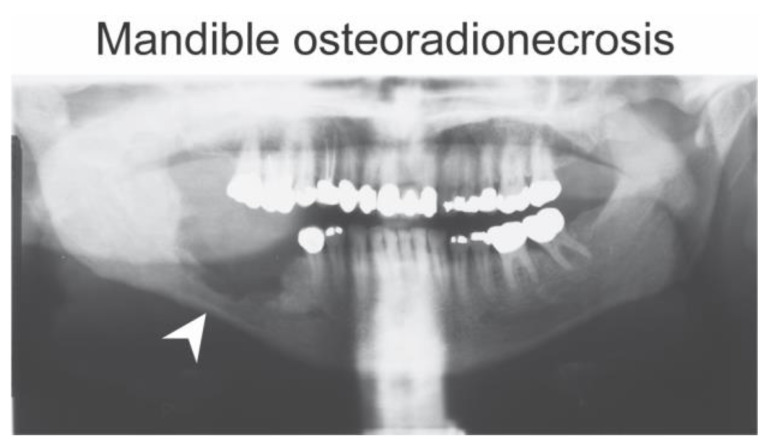
Mandible osteoradionecrosis. Panoramic radiograph of a head and neck cancer patient showing right mandible osteoradionecrosis (white arrowhead) that developed several months post-radiotherapy. The thin right mandibular cortical margin in the area of osteonecrosis poses a risk for potential pathological fracture of the mandible.

The number of cancer cases is projected to increase by more than 84% globally between the years 2022 and 2050 [1]. This includes head and neck cancers which are among the “Top 10” most common cancers. About 26% of head and neck cancer patients do not survive the first year after diagnosis due to cancer severity and treatment complications such as osteoradionecrosis (ORN) [2,3]. Reports on the incidence rates of jaw ORN are variable, ranging from 3.1% to 9.7% depending on the radiation dose and the duration of the radiotherapy [4,5]. This is an improvement from the previously high incidence rate of 44% prior to the advent of adequate pre-radiotherapy planning and patient preparations, dose fractionation, and the use of intensity-modulated radiation therapy (IMRT) [6,7]. A radiation dose greater than 50 Gy in the head and neck region makes the patient highly susceptible to ORN (Figure 1) [8,9]. Racial disparities also play a part in the incidence of ORN as both earlier and recent Surveillance, Epidemiology, and End Results (SEER) data showed that the 5-year relative survival rate for Caucasians with head and neck cancers was close to 57%. In comparison, African Americans had a lower survival rate, close to 33% [10,11]. Post-cancer therapy complications account for the majority of the disparities associated with poor cancer survival outcomes [10,11,12].

Although the exact relationship between race, head and neck cancers, and ORN is not fully understood [13,14,15,16], it is clear that these complications contribute to the poorer outcomes observed in patients of the Black race. Socioeconomic factors, access to specialized care, higher rates of alcohol and tobacco use, and potential biological differences all play a role in these disparities [17]. Studies have shown that Black patients with head and neck cancers have a higher frequency of mutations in key genes, such as P53 and FAT atypical cadherin 1 (FAT1), as well as fewer intra-tumoral effector immune cells [18]. Additionally, the increased copy number aberrations and miRNA-mediated silencing, like that seen with the proteoglycan 4 (PRG4) gene, further highlight the biological differences that may influence survival outcomes [19]. Addressing these disparities requires continued investigation into the molecular and clinical drivers of these differences, as well as efforts to improve access to high-quality care and reduce the socioeconomic barriers that disproportionately affect Black patients.

**Figure 2:**
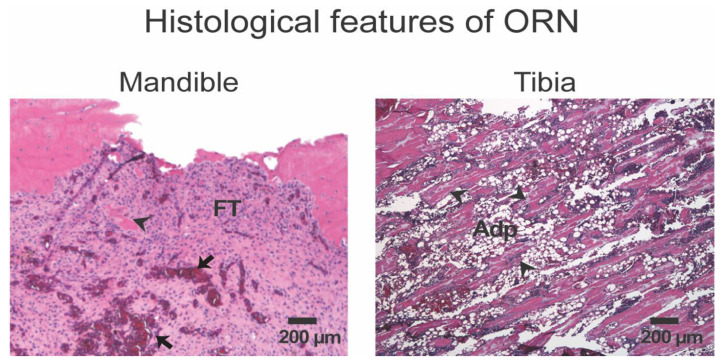
Comparative histological features of mandible and tibia osteoradionecrosis. Both the rat mandible and tibia received 50 Gy radiation. Histologically, the irradiated mandible displayed scanty acellular necrotic bone trabeculae (black arrowhead), tissue fibrosis (FT), and ruptured blood vessels (black arrow). The irradiated tibia displayed relatively more trabecular bone fragments (black arrowhead) and abundant adipocytic infiltration (Adp).

Head and neck cancer patients are susceptible to jaw ORN because the field of radiation during radiotherapy does not completely spare the jawbones [20,21]. Soft tissue fibrosis and trismus are early complications of cancer radiotherapy. ORN is a late complication of radiotherapy, but other factors like trauma or dental surgical procedures can hasten the development of ORN [8]. Clinically, jaw ORN can present as painless or painful non-healing bone exposure within the irradiated field. The histopathological features of ORN are based on radiation damage to the different bone cells and bone vasculature [22]. An ORN bone displays empty osteocyte lacunae, scanty mesenchymal stem cells, endothelial damage with extravasated red blood cells, and an irregular bone matrix caused by osteoclast resorption (Figure 2) [23]. Pathophysiologically, the onset and progression of ORN are theoretically attributed to the development of radiation-induced hypoxic-hypocellular-hypovascular tissue [22,24,25,26]. Additionally, the fibro-atrophic theory suggests that radiation-induced tissue inflammation, endothelial dysfunction, and fibrosis occur prior to frank bone breakdown during the process of ORN [27].

Compared to appendicular bones, the jawbones readily succumb to radiation damage. An animal study that directly compared similarly irradiated rat mandibles and tibias reported that early radiological and histological evidence of radiation damage occurred within 10 weeks in the mandible compared to 20 weeks in the tibia (Figure 2) [22]. Histologically, the mandible and tibia displayed the hypoxic-hypocellular-hypovascular features of ORN. While the mandible succumbed to 70% radiation-induced bone loss, the relatively structurally intact tibia displayed a trabecular microfracture and adipocytic marrow infiltrates [22]. These suggest the existence of regional skeletal site-specificity in the phenotypes and functions of the resident mesenchymal stem cells (MSCs) vital for bone homeostasis and repair following external insults like radiotherapy. Embryologically, craniofacial bones, including the jawbones, develop from migratory neural crest cells, while axial and appendicular bones develop from the mesoderm [28,29]. In humans and multiple animal species, orofacial MSCs are highly proliferative, with a longer population doubling capacity than the MSCs isolated from the non-oral skeletal sites [30,31]. Since highly proliferative cells are susceptible to radiation-induced DNA damage, the jawbones apparently develop ORN easily due to the cellular and local factors that may be unique to orofacial bones [32,33].

Pre-radiotherapy planning, early diagnosis of ORN, and prompt patient care after receiving radiotherapy are essential for ameliorating the effects of ORN [8]. There are still no definitive evidence-based therapies for ORN beyond preventive and palliative therapies or an invasive surgical resection that could complicate a patient’s quality of life. The rationale for this narrative review was to highlight the current diagnostic approaches to jaw ORN and the pathophysiological basis for new therapeutic options for ORN. A PubMed database search was performed without time limitation for the following Medical Subject Headings (MESHs) terms: osteoradionecrosis, jaw, therapies, radiation, diagnosis, and stem cells. A final search was performed on 28 June 2024. The article types were limited to the original studies and reviews in the English language and included both human and in vivo animal studies. Two investigators independently screened the articles and arrived at an agreement on the articles included in this review.

## 2. Risk Factors for Osteoradionecrosis of the Jaw

Risk factors for ORN in head and neck cancer patients receiving radiotherapy can be non-modifiable or modifiable and both significantly contribute to the onset of ORN. According to the guidelines from the International Society of Oral Oncology (ISOO), the Multinational Association of Supportive Care in Cancer (MASCC), and the American Society of Clinical Oncology (ASCO), the non-modifiable factors are those that cannot be changed through medical intervention or lifestyle modifications [8]. These factors include age, with older patients being more vulnerable due to the reduced healing capacity of aging tissues which directly impacts radiotherapy outcomes [8]. Biological sex also plays a role, as differences in susceptibility to ORN may exist between males and females, although the exact mechanisms remain unclear [8]. Additionally, a history of alcohol use significantly elevates the risk because alcohol impairs tissue recovery and ex-smokers still face a heightened risk due to the long-term effects of smoking on vascular and tissue health [34]. Tumor-related factors, such as the primary tumor location, T-stage, and nodal involvement, further influence the likelihood of ORN [8].

Comparatively, modifiable risk factors can be managed or addressed to reduce the risk of cancer complications. Poorly controlled diabetes mellitus is a major modifiable risk factor because high blood sugar levels impair wound healing and tissue repair [35,36]. A poor performance status, reflecting the patient’s overall health and ability to tolerate treatment, also increases the risk of ORN [8]. Pre-radiation dental evaluations play a critical role. The absence of a pre-treatment comprehensive dental assessment increases the likelihood of post-treatment complications. Similarly, failing to extract non-restorable teeth before radiation therapy significantly raises the risk of ORN [8]. The radiation technique used, along with dose–volume histogram parameters for the jaw, is another key factor in pre-treatment planning. The application of advanced techniques like intensity-modulated radiation therapy (IMRT) can limit the radiation to the jaw to reduce the risks [37]. Persistent smoking is one of the most critical modifiable factors. Active smokers face a substantially higher risk of developing ORN [8]. Chemotherapy, especially the use of platinum-based agents, has been recognized as a major risk factor for developing ORN. These drugs can intensify the harmful effects of radiation on bone tissue, making it more prone to necrosis. Interestingly, platinum-based chemotherapy in the absence of radiotherapy has been linked to an increased risk of osteonecrosis of the jaw [38]. Finally, the patients with severe or advanced periodontal disease are at greater risk due to the preexisting damage to the oral structures [34]. Addressing these modifiable factors is essential to mitigating the risk of ORN in patients undergoing radiation therapy.

## 3. Diagnostic Approaches to Osteoradionecrosis

The diagnosis and management of ORN are based on a patient’s history, as well as the clinical presentation combined with radiological and histopathological tests. There is a historical approach to the classification of ORN because several methodologies have been used to classify and grade ORN with each focusing on different aspects. The first well-known system, proposed by Robert Marx in 1983, categorized ORN based on how the patients responded to hyperbaric oxygen (HBO) therapy [25]. He described three stages: In the first stage, patients showed improvement after 30 dives of HBO, with full recovery after 60 dives [25]. The second stage included those patients that did not improve after 30 dives but improved after a sequestrectomy and additional HBO treatments. In the third stage, resection was performed if there was no improvement after both HBO therapy and a sequestrectomy. In the same year, Coffin introduced a classification system that divided ORN into minor and major forms depending on clinical and radiographic observations [39]. Minor ORN involved small bone fragments without radiographic evidence, while major ORN included significant bone damage visible both clinically and radiographically, sometimes accompanied by fractures [39]. A few years later, in 1986, Morton proposed another system that focused on the extent of the bone sequestration in ORN [40]. Minor ORN referred to the cases where the bone healed spontaneously, moderate ORN involved limited bone damage that healed in response to simple treatments, and major ORN was characterized by larger bone exposure and more severe symptoms, such as pathological fractures [40]. Epstein et al. (1987) developed a classification system based on the progression of the disease [41]. This classification identified three stages of ORN. Stage I was attributed to the ORN that resolved without a pathological fracture or with a pathological fracture that was reconstructed successfully [41]. Stage II was chronic persistent asymptomatic ORN with no evidence of clinical progression with or without a pathological fracture [41]. Stage III was active, progressive, and symptomatic ORN [41]. Glanzmann and Grätz, in 1995, proposed a system that focused on infection and treatment responses. This grading system ranged from ORN caused by an exposed bone with no infection (Grade 1) to death due to ORN (Grade 5) [42]. Lewis Clayman, in 1997, introduced a classification based on soft tissue involvement, with Type I involving intact tissue over the bone lesion, while Type II indicated tissue destruction and bone exposure [43]. In 2000, Store and Boysen classified ORN by the clinical and radiographic signs, starting with mucosal defects and a healthy bone (Stage 0) and ending with severe cases of fistulas and an exposed bone (Stage III) [44].

In 2002, Schwartz and Kagan developed a system centered on mandibular involvement, where Stage I referred to minimal ulceration, Stage II to more significant bone involvement, and Stage III to diffuse necrosis [45]. Notani et al. (2003) focused on the extent of bone damage, with Grade I associated with cases where the ORN was limited to the alveolar bone and with Grade III associated with cases of severe bone damage beyond the mandibular canal, often leading to bone fractures [46]. Tsai et al. (2013) introduced a treatment-based classification ranging from conservative measures (Grade 1) to major surgical interventions (Grade 4) [47]. Later, Karagozoglu et al. (2014) proposed a system based on how long the bone was exposed and whether there were any radiographic findings. Stage 0 referred to less than a month of bone exposure leading up to Stage III which involved extensive bone necrosis [48]. Lyons et al. (2014) also focused on how much bone was affected, ranging from less than 2.5 cm with no symptoms (Stage 1) to over 2.5 cm with fractures and other symptoms (Stage 4) [49]. He et al. (2015) introduced a system based on the size of the bone lesion and whether there were any soft tissue defects ranging from small lesions (Stage I) to fractures (Stage III) [50]. 

The most recent classification, released in 2024 by the American Society of Clinical Oncology (ASCO), is known as ClinRad. The system relies on objective clinical findings of exposed bone or the formation of a fistula and the vertical extent of the necrosis on imaging but does not include the presence of minor bony spicules in the classification [51]. ORN is classified into four stages. Stage 0 is characterized by radiographic evidence of bone necrosis that is confined to the alveolar bone with no clinical presentation (intact mucosa). This requires no intervention [51]. Stage I is the presence of clinical signs (primarily exposed bone) with or without radiographic evidence of ORN. Patients at this stage of ORN might require minor surgical intervention or medical management. Stage II is the radiographic evidence of bone necrosis that involves the basilar bone or maxillary sinus with or without clinical findings. This stage requires intermediate surgical intervention with or without medications. Stage III is characterized by advanced disease with the presence of one or more of the following features: a pathological fracture, oroantral communication, oronasal communication, or oro-cutaneous communication. At this stage, patients usually require reconstructive surgery with or without medications [51].

**Figure 3:**
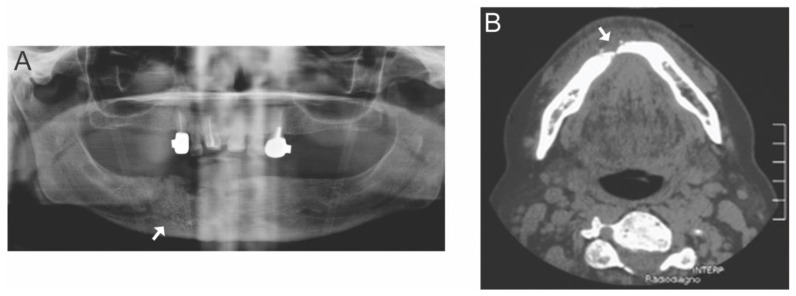
Dual diagnostic radiology of osteoradionecrosis. Right mandibular osteoradionecrosis was diagnosed with a combination of panoramic radiograph ((**A**), white arrow) and axial computerized tomography soft tissue window ((**B**), white arrow) at the level of mandibular body. (Images kindly provided by Dr. Mel Mupparapu, from the University of Pennsylvania, in Philadelphia, PA, USA).

Several radiographic imaging modalities are used for the diagnosis and evaluation of ORN. They offer the capability to qualitatively and quantitively assess the severity of ORN as well as the ability to monitor a patient’s response to treatment and overall prognosis. The commonly used imaging modalities include plain radiographs, computed tomography (CT), cone beam CT (CBCT), magnetic resonance imaging (MRI), and positron emission tomography/CT (PET/CT) [52]. Other imaging techniques used are multi-detector CT (MDCT), bone scintigraphy, and single-photon emission CT (SPECT) [53]. Plain radiography, such as panoramic radiographs, is mostly used in the initial evaluation of ORN [52]. Panoramic radiographic findings include ill-defined radiolucency with cortical thinning and destruction, an alteration of the architectural pattern of the trabecular bone, and a widening of the periodontal ligament spaces [52]. Computed tomography (CT) scans provide detailed images of bone and show the extent of bone destruction, bone sequestration, and bone fracture (Figure 3). CT combined with 18F-fluorodeoxyglucose positron emission tomography (PET/CT) is highly valuable in identifying the early stages of ORN, especially in at-risk patients [54]. PET/CT is particularly important for adequate patient follow-up when tumor recurrence is suspected [53]. Magnetic resonance imaging (MRI) is excellent for evaluating soft tissue involvement, marrow changes, and the extent of a necrotic lesion. It can detect early changes in the bone marrow that may not be visible on a CT and can more accurately assess the surrounding soft tissues [52]. Besides imaging, the use of blood-borne biomarkers has been proposed as an additional measure for diagnosing and evaluating the risks of ORN in susceptible patients [55,56,57,58]. These biomarkers include the pan-immune-inflammation value (platelet, monocyte, neutrophil, lymphocyte), hemoglobin to platelet ratio, hypoxia-inducible factors (HIF-1α), and vascular endothelial growth factor (VEGF) [56,57,58].

## 4. Therapeutic Approaches to Osteoradionecrosis

The management of ORN is challenging and complex. The treatment options differ according to the severity and duration of the disease [52,59]. In addition to the tumor and patient-related factors, cancer treatment modality plays a significant role in head and neck cancer survivorship [2]. The management of ORN ranges from surgical debridement and antibiotic therapy to more invasive flap surgery (Table 1) [9]. The conservative treatment options are local irrigation and debridement, systemic antibiotics, and the use of pentoxifylline with vitamin E [52,59]. The usual dose of pentoxifylline is 400 mg twice a day and the dose of vitamin E is 500 IU twice a day for 6 months or more [60,61]. Invasive surgical interventions are recommended when the bone damage and soft tissue damage are severe and extensive. The surgical procedures include a sequestrectomy, a maxillectomy/mandibulectomy with free flap reconstruction, and segmental resection [8].

**Figure 4:**
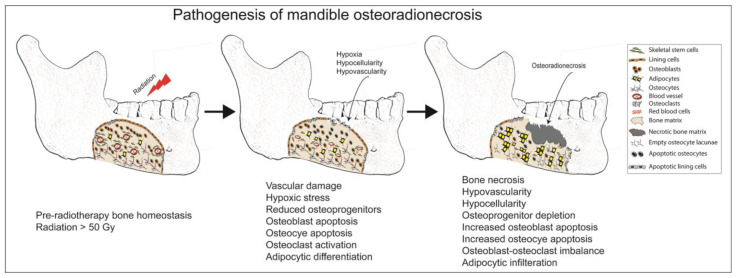
Summary of osteoradionecrosis pathogenesis.

HBO therapy can be used as well. However, its efficacy in the treatment and prevention of ORN is highly debatable [52,62,63]. HBO therapy is based on the hypoxic-hypocellular-hypovascular theory of ORN pathogenesis (Figure 4) [25]. HBO can be used as adjuvant therapy to promote tissue oxygenation and healing. While several HBO treatment protocols have been proposed, the clinical evidence in support of HBO therapy is limited and inconsistent. The use of HBO therapy is contraindicated in some patients with metastatic cancer because of the concerns that HBO can promote tumor revascularization [21]. Additionally, the cost of HBO therapy is prohibitively high, so it is sparingly used in most institutions.

## 5. Novel Therapies for Jaw Osteoradionecrosis

In addition to the existing management options, new treatments are being evaluated in clinical trials. The used of insulin-like growth factor I (IGF-I) is a promising treatment for ORN due to its role in bone proliferation and regeneration [64]. A study has shown that platelet-rich plasma effectively treated ORN when accompanied by surgery [65]. Stem cell therapy is also emerging as a potential treatment [59]. More research is needed to fully understand the efficacy and long-term outcomes of these treatments.

### 5.1. Mesenchymal Stem Cells (MSCs) in ORN Treatment

Creative approaches are needed to develop new novel therapies for ORN. Autologous bone grafts from non-oral sites, like the iliac crest and fibula, when used to reconstruct jawbone surgical defects, do not have clinical outcomes similar to site-specific donor grafts from a similar oral skeletal site [66,67]. This further supports regional skeletal site-specificity in the phenotypes and functions of the resident MSCs needed for bone homeostasis and repair following external insults such as radiotherapy. It is still unclear why donor jaw MSC grafts are more successful when the jaw is the recipient site. This is apparently related to the skeletal site-specificity of orofacial MSCs and the local factors unique to an orofacial bone [30,31,32,33].

The direct use of autologous MSCs as a potential treatment for ORN is still in development. The orofacial MSCs (OFMSCs) obtained from a jawbone as the donor site are functionally skeletal site-specific. Unlike non-orofacial skeletal sites such as axial and appendicular bones, OFMSCs are highly proliferative with more population doublings [31]. Despite the high proliferating property of OFMSCs, a sub-population of irradiated OFMSCs arrest during the G_0_G_1_ phase of the cell cycle but later recover from the damaging effects of irradiation to re-enter the cell cycle. Post-recovery, the OFMSCs can differentiate osteogenically to regenerate significant in vivo bone [32]. Thus, there is a potential that the interactions of the OFMSC donor graft with the residual MSCs in an irradiated jaw recipient site can be explored for new bone regeneration in ORN patients. There are still limited clinical studies on using MSCs for treating ORN because most reported studies focused on the animal models of ORN. The MSCs deposited into an ORN site will provide osteoprogenitor cells and growth factors that can initiate bone regeneration and healing. This is a therapeutic skeletal site-dependent adaptive ‘bystander’ effect of OFMSCs that has yet to be fully explored [32,68].

### 5.2. Insulin-like Growth Factor 1 (IGF-1) in ORN Treatment 

Osteoanabolic therapies such as those involving the administration of Insulin-like growth factor (IGF)-1, parathyroid hormone (PTH)1-34, and sclerostin-neutralizing antibody (Anti-SclMAb) are attractive therapies for ORN because they increase bone mass by stimulating bone formation while inhibiting bone resorption [69]. They have also shown promising results in promoting healing and new bone formation in a radiation-damaged bone [69,70,71]. Unfortunately, the parenteral delivery of these therapies undermines patient compliance and treatment outcomes. IGF-1 facilitates the survival, proliferation, and differentiation of bone-forming osteoblasts and bone-resorbing osteoclasts to regulate bone homeostasis [64]. The local delivery of IGF-1 accelerated bone formation in a rat fracture healing model [72], and IGF-1 administration in patients increased bone healing, with rapid clinical improvements in hip or tibial fractures during osteoporosis [64]. These IGF-1 functions in bone growth and remodeling enhance the potential for the oral delivery of IGF-1 as a therapy for multiple skeletal disorders. The promising outcomes of early studies emphasize the need for well-designed clinical trials on the therapeutic use of IGF-1 for jaw ORN.

### 5.3. Parathyroid Hormone (PTH) in ORN Treatment

PTH is commonly used to treat osteoporosis because it acts on osteoblasts to stimulate bone synthesis. Clinically, the dosing regimen is a daily subcutaneous injection of 2 µg in the thigh or abdomen [73]. In animal studies, the protective effect of PTH from radiation-induced bone damage has been well reported [71]. PTH(1-34) treatment prevented osteoblast and osteocyte apoptosis, decreased the adipocytic differentiation of marrow stromal elements, and reversed the damaging effect of radiation [71]. The ability of PTH to reverse radiation-induced damage was attributed to WNT-pathway-induced DNA repair in the osteogenic cells [74]. Despite numerous animal studies on the PTH treatment of radiation bone damage, reports on the use of PTH for ORN treatment in humans are still limited [75]. The ORN-treated patients with improved clinical outcomes required 6 months of daily treatment and there are indications that PTH therapy may be better suited for refractory cases of ORN. Despite the similar use of PTH for the treatment of medication-related osteonecrosis of the jaw (MRONJ) [76], there are still concerns about the risks of PTH-induced osteosarcoma based on the animal studies that reported the development of osteosarcoma in rats [75]. However, these osteosarcoma findings have not translated to similar outcomes in humans. [77,78]. While the animal studies on PTH treatment for ORN are promising, more studies are needed to conclusively evaluate the clinical evidence in support of PTH use in ORN management. The rational use of PTH treatment for jawbone necrosis is still challenged by patient compliance. The cost, the long-term treatment of up to 6 months, the periodic monitoring of bone turnover markers, and vitamin D supplementation are the factors that dampen patient compliance.

## 6. Conclusions

In summary, ORN is still a recalcitrant skeletal complication of orofacial cancer radiotherapy. The anatomical, skeletal, cellular, and molecular processes that make the jawbone highly susceptible to ORN are still unclear but continue to evolve based on animal and clinical studies’ findings. There are still no definitive non-surgical therapies for ORN, but future clinical studies on osteoanabolic therapies for ORN may yield promising results that can be translated into clinical practice. The new outcomes should increase the diagnostic and therapeutic options for jaw osteoradionecrosis.

## Figures and Tables

**Table 1:** Management of osteoradionecrosis.

	Osteoradionecrosis Therapy	Advantages	Disadvantages
1	Conservative management Local irrigation Debridement Systemic antibiotics	For asymptomatic or mildly symptomatic patients, with early or moderate disease Simple, convenient	Poor outcomes with 15% cure rate Need to be combined with other management options Risks of lesion progression
2	Hyperbaric oxygen therapy (HBO)	Improves angiogenesis Bactericidal or bacteriostatic effects Used in prevention and treatment of ORN	Not applicable in advanced or refractory cases of ORN Not applicable when residual tumor tissue is suspected Expensive treatment An adjunctive treatment method
3	Medical treatment options Tocopherol (TCP) Denosumab Pentoxifylline (PTX) Clodronate (CLO)	Simple, convenient Low cost	No demonstrated effect on resolution of ORN when used alone
4	Surgery	For advanced ORN Surgery was the only treatment option available, if conservative measures and HBO therapy failed to control ORN	Complications
5	Ultrasound therapy	Induces angiogenesis Improves blood flow to muscles	Mechanism of action unclear Ultrasound treatment parameters are unclear
6	Mesenchymal stem cell therapy	Induces bone regeneration Complete ORN without side effects	Early stages of development for ORN therapy Limited ORN clinical studies Expensive and not readily available
7	Osteoanabolic therapy Insulin-like growth factor 1 (IGF-1) Parathyroid hormone (PTH)	IGF-1 promotes marrow stromal cell differentiation and osteoblast proliferation IGF-1 induces bone regeneration PTH prevents osteoblast and osteocyte apoptosis, decreases adipocytic differentiation of marrow stromal elements PTH osteoclastic effect promotes bone remodeling	Early stages of development for ORN therapy Systemic delivery only Limited ORN clinical studies
